# A closed-loop tray bioreactor for online monitoring of mycelium-based composites

**DOI:** 10.3389/fmicb.2026.1877723

**Published:** 2026-09-23

**Authors:** Tiziano Derme, Che Wei Lin, Francis Willis Mathew Robert Schwarze, Benjamin Dillenburger

**Affiliations:** 1 ITA-Institut für Technologie in der Architektur, ETH Zürich, Zürich, Switzerland; 2 Empa Cellulose & Wood Materials Laboratory, St. Gallen, Switzerland

**Keywords:** growth kinetics, mycelium-based composites, respirometry, solid-state fermentation, tray bioreactors

## Abstract

Mycelium-based composites are bio-derived structural materials produced by cultivating filamentous fungi through lignocellulosic substrates under solid-state fermentation conditions. Solid-state fermentation remains difficult to control because fungal growth is highly sensitive to local gradients in temperature, humidity, and gas composition. This study presents the design, construction, and experimental validation of a modular tray bioreactor for the controlled cultivation of mycelium-based composites. The system combines closed-loop feedback control of temperature and relative humidity with continuous monitoring of carbon dioxide and oxygen, online calculation of carbon dioxide evolution rate, programmable environmental trajectories, and synchronized multi-unit operation through a primary-secondary architecture. Performance was evaluated in two abiotic and two biotic experiments. Under abiotic conditions, the reactor provided stable regulation within a practical operating range of approximately 26–29°C and 25–50% relative humidity, with a steady-state temperature error below 0.5°C at the established setpoints and a root mean square error of 0.12°C during fixed-set-point operation. During biotic experiments with *Ganoderma sessile*, environmental regulation remained comparable, while carbon dioxide accumulation and carbon dioxide evolution rate clearly distinguished biological activity from the abiotic baseline and resolved the transition from internal growth in the closed mould to external growth after demoulding. The biotic runs (one dynamic and one constant schedule, conducted without biological replicates) are presented as a proof-of-concept demonstration of the platform rather than a quantitative study of growth kinetics, and the operating envelope and the limited resolution of the electrochemical oxygen sensor for OUR and RQ analysis are reported as current hardware constraints. The platform was also deployed as a synchronized four-unit array across chambers of different sizes. Together, these results establish a reproducible experimental platform for controlled fermentation of mycelium-based composites and for future studies linking environmental regulation with growth dynamics and material performance.

## 1 Introduction

Mycelium-based composites (MBCs) are bio-derived structural materials produced by cultivating filamentous fungi through lignocellulosic substrates under solid-state fermentation (SSF) conditions (Yoon et al., 2014). As the colony develops, a three-dimensional self-bonded network of chitin-rich hyphae permeates and binds the substrate particles, producing a bio-composite that can be grown into precise and complex geometries by mold confinement during fermentation (Raimbault, 1998; Betts et al., 2019; Bayer and Mcintyre, 2010). The resulting material combines low bulk density (typically 50 -200 kg m^–3^), adequate compressive and flexural strength for low-load packaging and structural applications, competitive thermal insulation performance, and complete aerobic biodegradability within weeks under composting conditions (Jones et al., 2020; Delabona et al., 2013; Holt et al., 2012; Elsacker et al., 2019). These properties have attracted substantial academic and industrial interest in MBCs as a replacement for expanded polystyrene and polyurethane foams, and commercial production has been demonstrated at scale. Nevertheless, the transition from laboratory protocols to reliable equivalents on an industrial-scale remains difficult and underdeveloped, with the design and engineering of a climatically controlled fermentation environment and the understanding of SSF kinetics identified as the main limiting factors (Derme et al., 2024; Rodríguez-Leoń et al., 2018). The central engineering challenge of the process is that the formation of MBCs is a solid-state process, and SSF is inherently difficult to control and monitor (Simutis and Lübbert, 2015). Unlike submerged liquid fermentation, SSF occurs in the near-absence of free water, thereby eliminating the convective mixing and heat dissipation mechanisms that make liquid-phase bio-processes tractable. Fungal biomass generates substantial metabolic heat during active colonization, estimated at 40–120 W per kg of dry substrate. This heat accumulates within the substrate mass and creates steep spatial gradients in temperature, oxygen availability (O_2_), and carbon dioxide concentration (CO_2_) (Saucedo-Castañeda et al., 1990). These gradients translate directly into heterogeneous colonization: the peripheral regions of the substrate are over-aerated and rapidly desiccated, while the interior regions become anoxic and heat-stressed (Singhania et al., 2007). Because the mechanical properties of the final composite are determined by the spatial density and continuity of the mycelial network, uncontrolled SSF produces parts with non-uniform density, anisotropic mechanical behavior, and high batch-to-batch variability — outcomes that are incompatible with quality-assured manufacturing. Tray bioreactors are the natural configuration for the production of MBCs on an industrial scale, as the absence of mechanical agitation preserves the structural integrity of the developing hyphal scaffold (Chen, 2013; Wang et al., 2018; Kaplan-Bie et al., 2019). Forced air convection substantially improves heat and mass transfer over static operation but introduces a critical variable coupling problem. Increasing airflow simultaneously reduces CO_2_ partial pressure, accelerates evaporative moisture loss from the substrate surface, and increases O_2_ supply, each of which directly affects the kinetics of fungal growth and therefore the metabolic heat generation rate itself. Quantitative studies in instrumented SSF tray systems confirm that increasing the Reynolds number of the head-space significantly increases both the heat and the mass transfer coefficients, but proportionally increases the loss of substrate water (Figueroa-Montero et al., 2011). At the process level, high aeration rates under automated humidity control have been shown to produce net negative effects on yield due to progressive desiccation, confirming that airflow and humidity cannot be adjusted independently (Farinas et al., 2011). Adjusting the inlet air temperature further shifts the saturation vapor pressure and therefore the effective relative humidity independently of any humidity setpoint (Figueroa-Montero et al., 2011). These interdependencies mean that single-input single-output control strategies produce persistent cross-variable disturbances, and multiple-input multiple-output (MIMO) hardware and control systems are required to achieve adequate regulation of coupled multi-variable bio processes (Hausmann et al., 2017). Within the MBCs field, production has been conducted almost exclusively in static trays or open mold incubation chambers. The closest published approach specifies targets for passive CO_2_ accumulation within a sealed enclosure but does not provide active sensor-driven feedback or disturbance-rejection capability (Ross et al., 2023; Kırdok et al., 2022). The consequences of uncontrolled environmental deviations are particularly severe for MBCs production because the resulting damage is irreversible and spatially localized. Temperature excursions above the species-specific vegetative growth optimum redirect metabolic resources toward sporulation and permanently affect colonization in the affected substrate region (Grimm et al., 2005; Rahardjo et al., 2006). CO_2_ accumulation above inhibitory thresholds suppresses the density of the branching of the hyphae, reducing the mechanical isotropy of the final compound; online respirometric monitoring has established that a respiratory quotient (RQ) exceeding unity is a diagnostic of the formation of anaerobic zones and inadequate gas exchange within the fermentation bed (Torres-Mancera et al., 2018). Substrate desiccation below the critical water activity threshold arrests hyphal extension irreversibly in the desiccated zone. Because the mechanical performance of the composite material is determined by the spatial continuity of the hyphal network throughout the volume of the substrate, any localized environmental deviation produces a permanent material defect that cannot be corrected. No published MBC study has demonstrated simultaneous closed-loop control of all primary SSF process variables. This study presents the design, construction, and experimental validation of a modular closed-loop tray bioreactor for the solid-state fermentation of mycelium-based composites. The system provides independent PID feedback control of temperature and relative humidity, open-loop airflow regulation based on a pre-characterized fan-speed calibration, and continuous monitoring CO_2_ and O_2_ for real-time respirometric estimation of fungal activity. The instrumented lid is physically decoupled from the reactor enclosure, allowing the same control assembly to be deployed across chambers of different sizes; in this study, two standard glass enclosures 1,000 × 500 × 500 mm (L × W × H) and 1,000 × 1,000 × 500 mm (L × W × H) were evaluated. The platform also supports custom time-varying environmental profiles and multi-unit operation through primary-secondary units synchronization over TCP/IP for coordinated setpoints and incubation timing. The performance of the controller was experimentally characterized under both abiotic and live fermentation conditions. The primary contribution of this work is a reproducible experimental platform for solid-state MBC fermentation that enables a systematic study of process–structure–property relationships under precisely defined and actively regulated environmental conditions (Figure 1).

**FIGURE 1:**
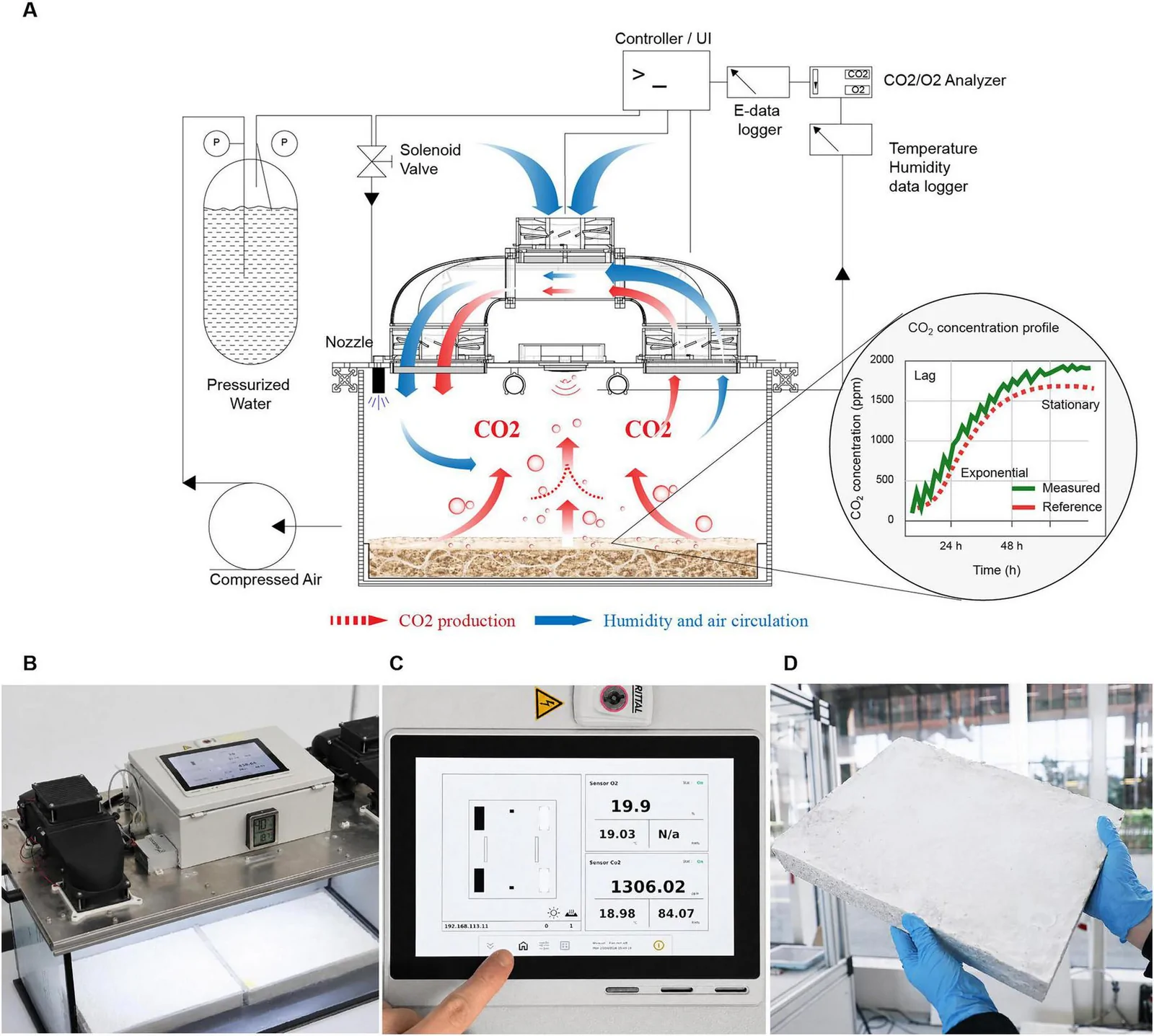
**(A)** Operating schematic of the closed-loop cultivation chamber, showing the pressurized-water humidification line, recirculating airflow through the instrumented lid, head-space sensing and exhaust-gas analysis, and the generation of CO_2_ within the substrate tray. The inset shows an illustrative (schematic) CO_2_ accumulation profile over a fermentation, indicating the expected lag, exponential, and stationary phases; it is conceptual and does not represent measured data or an independent reference measurement. **(B)** Physical prototype with enclosure, substrate tray, tubing, and sensor-integrated lid. **(C)** Local touchscreen interface used for process visualization and setpoint adjustment. **(D)** Representative mycelium-based composite cultivated in the reactor.

## 2 Materials and methods

### 2.1 Bioreactor and hardware design

The bioreactor comprises three components: an (i) instrumented lid, (ii) interchangeable enclosure, and (iii) a standalone control unit. The lid consolidates all sensors, air circulation fans, humidification hardware, and 3D-printed air duct assemblies into a single assembly that fits into the enclosure with a gas-tight seal. This modular architecture decouples the instrumentation from the reactor volume, allowing the same lid to be deployed across enclosures of different sizes without modification. Two standard glass enclosures were evaluated: 1,000 × 500 × 500 mm (L × W × H) and 1,000 × 1,000 × 500 mm (L × W × H). A custom-fabricated substrate tray is positioned within the enclosure base to accommodate the mold-confined geometry required for near-net-shaped composite production. Internal air is recirculated by fans mounted on the lid. HEPA filters are installed at all three air interfaces: recirculation supply inlet, fresh air exchange inlet, and exhaust outlet. These components provide independent biological containment at each point. The inlet air temperature is regulated by a 100 W 12 V positive-temperature-coefficient (PTC) heater integrated into the lid airflow circuit; the heater power is continuously modulated via pulse-width-modulation (PWM) under closed-loop temperature feedback. Humidification is achieved through an ultrasonic atomizing nozzle supplied by a pressurized water tank, with delivery controlled by a solenoid valve. Fresh air is admitted at an operator-defined exchange rate (air changes per hour), which governs steady-state CO_2_ concentration; The exhaust gas is routed to the CO_2_/O_2_ analyzer for respirometric measurement. The bioreactor is designed to support future closed-loop CO_2_ control through automated actuation of the fresh air inlet. The control unit provides a touchscreen interface for local operation and a web interface for remote monitoring and setpoint adjustment.

### 2.2 Sensors and instrumentation

Two sensor assemblies were mounted on the underside of the instrumented lid at opposite ends of the chamber. An assembly comprised a DFRobot Gravity electrochemical oxygen sensor (0%–25%) vol., I_2_C), which provided continuous in situ measurement of O_2_ concentration and local chamber temperature. The second assembly comprised a Seeed Studio SCD30 module (Sensirion SCD30, I_2_C), which measured CO_2_ by non-dispersive infrared absorption, relative humidity by capacitive sensing, and temperature. Positioning the temperature sensors at opposite ends of the enclosure enabled detection of spatial gradients across the substrate surface during active colonization. All sensor signals were acquired through I_2_C and logged continuously by the control unit. Volumetric airflow through the recirculation circuit was characterized separately using a calibrated thermal anemometer (PCE-423N, PCE Instruments) at the exhaust of the recirculation HEPA filter. This one-time calibration established the relationship between fan speed and airflow used for subsequent open-loop airflow regulation; the anemometer was not used as a continuous in-process sensor (complete calibration procedure in Supplementary Methods 1.2).

### 2.3 Control system

The control system was implemented on a Seeed Studio reTerminal incorporating a Raspberry Pi Compute Module 4 (CM4) running the Raspberry Pi OS. The CM4 served as the central platform for sensor polling, feedback control, data logging, and operator interaction. Sensor communication was handled over I_2_C and control logic was implemented in Python. Two independent PID control loops, with tuned gains reported in Supplementary Table 1 and the tuning procedure described in Supplementary Methods 1.1, operated continuously in parallel. The temperature controller used the mean of the two in-chamber temperature measurements to modulate the PTC heater via pulse-width modulation and maintain the temperature setpoint. The humidity controller used the SCD30 relative humidity measurement to actuate the humidification solenoid valve. Airflow was regulated in open loop through the pre-characterized fan speed-to-flow calibration, while fresh-air exchange was set by the operator to determine the overall gas exchange rate. Continuous sensing of CO_2_ and O_2_ provided real-time monitoring of head-space composition and supported online respirometric calculations. The hardware and software architecture were designed to support future extension to closed-loop airflow and gas-composition control. The operator interface was implemented using LVGL (v8) and rendered locally on the reTerminal touchscreen, with mirrored access through a web interface on the local network. All sensor readings and control outputs were logged continuously in structured files in CM4. The software also supported multi-unit operation through a primary-secondary units synchronization over TCP/IP, in which one unit broadcast setpoints, elapsed time and recipe stage to connected secondary units; the message format and fallback behavior of the synchronization protocol are described in Supplementary Methods 1.4 (Figure 2).

**FIGURE 2:**
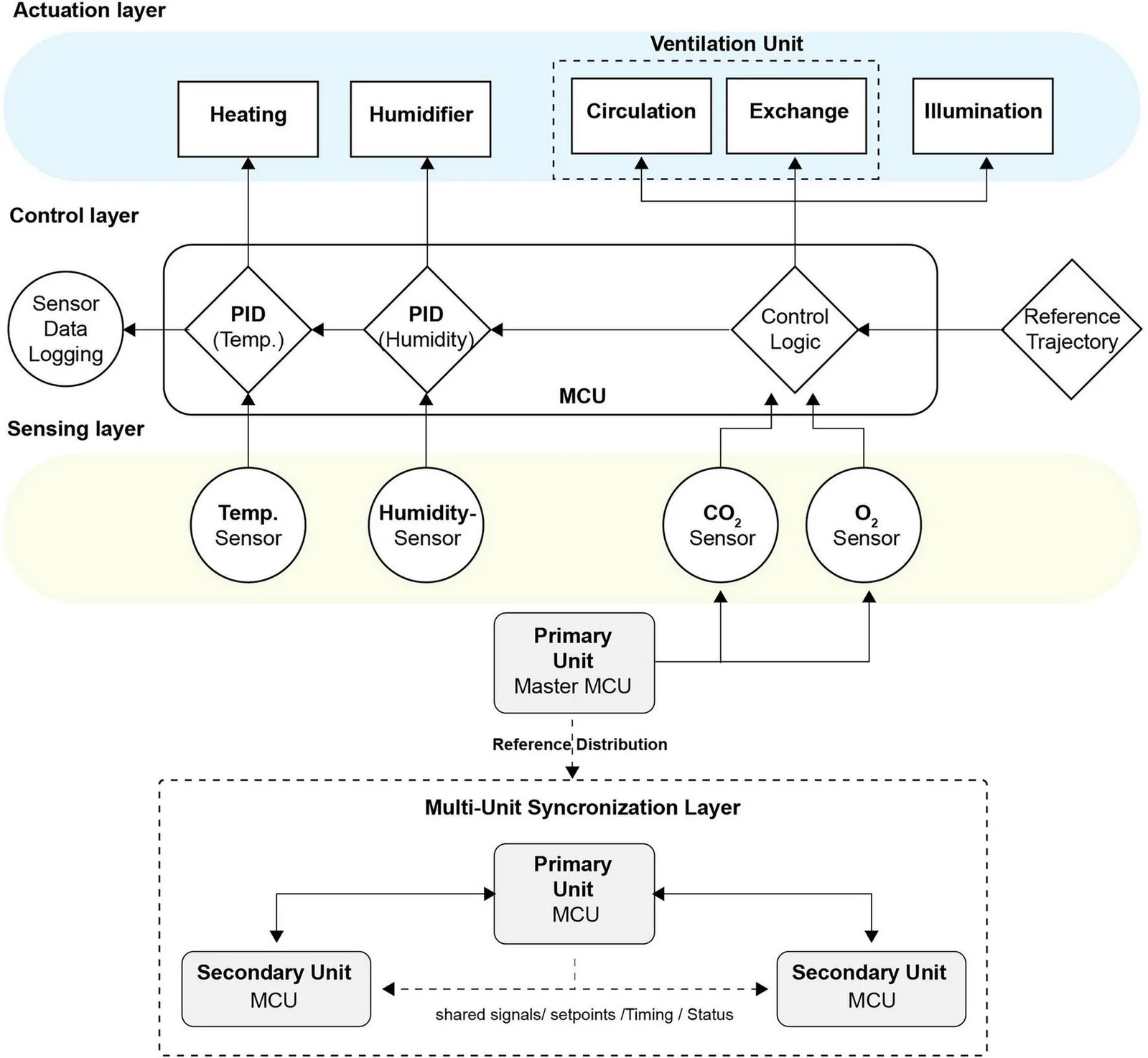
Block diagram of the closed-loop control architecture for the mycelium bioreactor. The MCU receives feedback from temperature, humidity, CO_2_, and O_2_ sensors and actuates the heating, humidification, and ventilation subsystems to maintain the desired cultivation environment. Temperature and humidity are regulated by PID controllers, while gas-related inputs are processed by supervisory control logic driven by a reference trajectory. Sensor data are continuously logged, and a synchronization layer enables a primary unit to distribute reference signals and operating sequences to secondary bioreactor units.

### 2.4 Respirometry and online growth rate estimation

Fungal metabolic activity was characterized in real time throughout each fermentation by continuous measurement of CO_2_ and O_2_ concentrations in the chamber head-space using the SCD30 and DFRobot sensors described in section “2.2 Sensors and instrumentation.” The head-space was treated as a continuously stirred tank reactor (CSTR) with respect to the gas phase: internal recirculation by the lid fans homogenizes the head-space atmosphere but does not contribute to net accumulation or depletion of CO_2_ or O_2_, since recirculation neither adds nor removes gas. The only net exchange with the external atmosphere occurs through the fresh-air inlet at the volumetric flow rate F_*a*_ (m^3^ h^–1^), determined from the air-exchange rate set by the operator and the known enclosure volume. The inlet composition was taken as constant at standard atmospheric values (y_*CO*_,_*atm*_ = 4.0 × 10^–4^; y_*O*_,_*atm*_ = 0.2095), valid given the absence of any active gas supply and the continuous admission of unmodified atmospheric air. Under these conditions, the CO_2_ evolution rate (CER) and O_2_ uptake rate (OUR), each normalized to the dry substrate mass, are given by Torres-Mancera et al. (2018):


$$\text{CER}=\frac{\text{Fa}\,\left(y\,{\mathrm{CO}}_{2},\mathrm{hs}-y\,{\mathrm{CO}}_{2},\mathrm{atm}\right)}{V\,\text{e}}\cdot \frac{1}{\rho \,\text{s}}$$ (1)

$$\mathrm{OUR}=\frac{\text{Fa}\,\left({\mathrm{yO}}_{2},\mathrm{atm}-{\mathrm{yO}}_{2},\mathrm{hs}\right)}{\text{Ve}}\cdot \frac{1}{\rho \,\text{s}}$$ (2)

where *yCO*_2_,hs and *yO*_2_,hs, are the real-time head-space mole fractions measured by the SCD30 and DFRobot sensors, *V*_*e*_ is the head-space volume (m^3^), and ρ_*s*_ is the dry substrate mass (kg). The respiratory quotient was defined at each sampling interval as:


$$R\,Q=\frac{\text{CER}}{\text{OUR}}$$ (3)

In this study, CER was the only respirometric quantity computed from valid real-time data and used for quantitative interpretation. Although the control software implements Equations 1–3 continuously, the 0.1% vol. quantization of the electrochemical O_2_ cell exceeded the metabolic O_2_ depletion observed under the present operating conditions, as shown in Supplementary Figure 1, so OUR and RQ could not be resolved reliably and are reported only as raw traces. An RQ consistently exceeding unity is, in principle, a diagnostic indicator of inadequate gas exchange and the onset of localized anaerobic conditions within the substrate bed; this readout becomes available once O_2_ sensing of sufficient resolution is implemented (section “4.3 Technical limitations and interpretation boundaries”). Estimation of the instantaneous specific growth rate (μ) from OUR additionally requires a strain-specific biomass-on-oxygen yield coefficient (*Y*_*O*2_) and an estimate of biomass concentration (X); because no *Y*_*O*2_ has been reported for *Ganoderma sessile* under solid-state fermentation, and reported values for other filamentous fungi vary several-fold and are not constant over a run, the μ framework is presented in Supplementary Methods 1.5 and treated as a target for future work rather than evaluated here. Implementation details, including the noise-filtering window and per-chamber head-space volumes used in Equation 1, are reported in Supplementary Methods 1.5.

### 2.5 Biological materials and fermentation protocol

The fungal strain used in this study was *G. sessile*, isolated from fresh fruiting bodies by tissue culture and kept on malt extract agar before spawning preparation. *G. sessile* was selected because it is representative of *Ganoderma* strains used in the production of commercial mycelium-based material, including by companies such as Ecovative and Mogu. The species produces annual, typically sessile basidiocarps in living or dead hardwoods, is associated with root and butt rot in hosts such as *Quercus*, *Acer*, and *Fagus*, and forms abundant chlamydospores in culture (Murrill, 1908). It is currently treated as synonymous with *Ganoderma resinaceum* in some classifications and is widely distributed throughout eastern North America (Bazzalo and Wright, 1982; Steyaert, 1980). The grain spawn was prepared on sterilized millet grain and used as inoculum at 16% (w/w) relative to the dry mass of the substrate. The substrate consisted of hemp shives with a maximum particle length of 4 mm, supplemented with wheat bran [5% (w/w)] as an additional source of carbon and nitrogen, and gypsum [1% (w/w)] as a pH buffer and structural conditioner. The moisture content of the substrate was adjusted to 62% (w/w) before sterilization. All substrate batches were sterilized by autoclaving at 121 °C for 15 min at 103 kPa, then cooled to room temperature under sterile conditions before inoculation. The inoculation was performed aseptically by thoroughly mixing the grain spawn with the cooled substrate at the target rate. The inoculated substrate was transferred to polypropylene bags and incubated at approximately 26 °C and 60% relative humidity for 5 days to allow initial colonization before transfer to the bioreactor. Following this pre-incubation stage, the partially colonized substrate was placed in the custom-fabricated polypropylene (PP) vacuum-formed tray and sealed within the bioreactor enclosure beneath the instrumented lid. The material-formation protocol comprised two sequential phases distinguished by the state of the mold. During the internal-growth (closed-mold) phase, the inoculated substrate was confined within the custom PP vacuum-formed tray beneath the instrumented lid. As colonization proceeded, the developing hyphal network bound the substrate particles into a coherent, self-supporting body in the net shape of the mold; this hyphal binding is initially temporary and provides the mechanical integrity required for the subsequent step. After 2.8 days, the consolidated part was removed from the mold (containing boundary) but retained within the same instrumented chamber, exposing the previously confined surfaces to the chamber head-space and initiating the external-growth (exposed) phase, during which the fungus formed a denser, continuous mycelial skin over the exposed surfaces. Because the part remained in the chamber throughout, head-space gas composition was recorded continuously across the transition. The two phases therefore correspond to bulk consolidation of the composite within the mold (internal) followed by surface-skin formation after demoulding (external), and produce the distinct surface textures shown in Figure 4E: a substrate-exposed fibrous texture after the closed-mold phase, and a smoother, denser mycelial skin after the exposed phase. Extended substrate preparation and pre-incubation details are provided in Supplementary Methods 1.3.

### 2.6 Experimental design for controller validation

The performance of the controller was evaluated through four experiments: two abiotic runs conducted without biological material, followed by two biotic runs using an inoculated substrate prepared as described in section “2.5 Biological materials and fermentation protocol.” The abiotic experiments were used to determine the controllable operating range of the bioreactor and to establish the baseline of the gas-phase in the absence of metabolism, while the biotic experiments were used to assess environmental regulation during live fermentation and to demonstrate online physiological monitoring. The first abiotic experiment consisted of a 48 h multi-step temperature–humidity schedule designed to probe controller performance across a range of operating conditions. The programmed trajectory included successive changes at the temperature and relative humidity set points that approximate a circadian-like profile, followed by a terminal stress segment at elevated temperature and reduced relative humidity. The second abiotic experiment consisted of a fixed-set-point run held at 25 °C and 45% RH. In both abiotic runs, performance was assessed from tracked temperature and relative-humidity signals using steady-state error, root mean square error (RMSE) and qualitative evaluation of settling behavior. Head-space CO_2_ and O_2_ were continuously recorded to confirm atmospheric baseline behavior under non-biological conditions. The two biotic experiments followed the same comparative logic under live fermentation conditions. In both cases, a partially colonized substrate was incubated in the bioreactor over a 4.5-day material formation protocol that included a 2.8-day internal-growth phase in the closed mold, followed by a 1.7-day external-growth phase after opening the mold. In the first biotic run, a multi-step circadian-plus-stress schedule derived from the abiotic profile was extended across the 4.5 days process (multi-step run). In the second biotic run, constant conditions of 25 °C and 45% RH were maintained throughout the usable recording window (fixed-set-point). Environmental regulation was evaluated from the tracked temperature and relative-humidity profiles, while physiological activity was assessed from continuous head-space gas measurements. The CER was continuously computed according to Equation 1 on page 1, and phase-specific metrics were compared between internal and external growth. OUR and respiratory quotient were not used for quantitative interpretation because the resolution of the electrochemical sensor O_2_ was insufficient under the present fermentation conditions.

## 3 Results

The results are presented in three parts: abiotic controller validation, biotic controller validation with online physiological monitoring, and multi-unit synchronization in an array configuration.

### 3.1 Abiotic controller validation

The controller was first assessed in non-biological environments to set a baseline to track performance, ensuring that biological factors did not interfere with the results (Table 1). Two experiments were conducted: a 48-h multi-step temperature–humidity schedule that approximates circadian variation and ends in a terminal stress segment, and a fixed set-point at 25 °C and 45% RH. In both experiments, the temperature controller used the SCD30 head-space sensor (T2) as the feedback signal, while the electrochemical O_2_ sensor assembly (T1) was recorded for spatial diagnostics only (Figure 3). During the multi-step run, T2 followed the programmed trajectory across the segments whose setpoints lay within the controllable range 26.5 °C-28.0 °C (Figure 3A). Across these segments, steady-state error remained below 0.5 °C, RMSE ranged from 0.13 °C to 0.46 °C, and standard deviation within settled intervals was 0.03 °C-0.07 °C. The re-establishment of each new setpoint occurred within approximately 30 min. full per-segment metrics, including the transient portion of each step are reported in Supplementary Table 2. Comparable performance was observed during the fixed-set-point run (Figure 3E, where T2 stabilized at 24.93 °C ± 0.11 °C against the 25 °C target, corresponding to a steady-state error of −0.08 °C and an RMSE of 0.12 °C, over the full run. By contrast, when scheduled temperatures fell below approximately 26 °C, T2 remained 1.4 °C-2.7 °C above setpoint, indicating a lower operating limit under the present hardware configuration. Relative humidity showed a similar pattern of accurate tracking within the controllable range and saturation at the upper boundary. For segments with setpoints for RH ≤ 40%, the tracking error remained low, with RMSE values of 0.24%–0.80% RH and standard deviations below 1.3% RH. However, at higher setpoints (≥51%), RH stabilized 11%–16% below the target and reached a practical ceiling of approximately 47%–50% RH (Figure 3B). The fixed-set-point experiment confirmed the same upper limit: at a 45% RH target, the controller stabilized at 46.19 ± 1.64% RH with an RMSE of 2.03% (Figure 3F). Together, these results define the operating humidity range achieved under the airflow conditions used here. In the terminal stress segment, the controller was set at 30 °C and 25% RH. T2 approached the target trajectory but reached only 28.7 °C at the end of the 48-h run, corresponding to a steady-state error of −1.3 °C, while RH remained close to the low-humidity target 29.0 ± 0.12% (Figures 3A, B). In parallel, head-space CO_2_ and O_2_ remained close to atmospheric values throughout both abiotic experiments (Figures 3C, D, G, H). In the multi-step run, CO_2_ fluctuated between 365 and 415 ppm without sustained accumulation, while O_2_ remained within 20.2%–20.7% (v/v). In the fixed-set-point run, CO_2_ decreased from an initial elevated value of approximately 470 ppm to 383 ± 5 ppm in about 2 h and remained stable thereafter. The O_2_ signal showed a systematic offset of approximately 0.5% below the theoretical atmospheric value. A summary of abiotic gas-phase statistics is provided in Supplementary Table 3. Overall, abiotic experiments show that the bioreactor provides stable closed-loop regulation of temperature and relative humidity within a defined operating range, while head-space gas composition remains near atmospheric equilibrium in the absence of metabolism. Under the airflow configuration used here, this range was approximately 26 °C -29 °C and 25%–50% RH, with deviations outside these limits reflecting hardware constraints rather than unstable control behavior. Therefore, these abiotic measurements establish the baseline for the interpretation of the live-fermentation data presented in section “3.2 Biotic controller validation and online growth monitoring.”

**TABLE 1:** Per-segment controller tracking metrics for the two abiotic experiments.

Segment	T setpoint (°C)	RH setpoint (%)	T_2_ mean ± SD (°C)	T_2_ RMSE (°C)	RH mean ± SD (%)	RH RMSE (%)
Multi-step (48 h)						
1–15 h	27.0	40.0	26.67 ± 0.37	0.49	39.37 ± 1.46	1.59
15–18 h	28.0	34.0	27.60 ± 0.07	0.12	34.20 ± 0.28	0.35
18–27 h	26.5	38.0	26.38 ± 0.05	0.13	37.65 ± 1.11	2.60
27–39 h	24.5	48.0	24.55 ± 0.14	0.15	47.12 ± 1.84	2.04
39–48 h	28.0	30.0	28.02 ± 0.70	0.70	31.69 ± 2.71	3.19
Fixed-set-point (24 h)						
0–24 h	25.0	45.0	24.93 ± 0.10	0.12	46.19 ± 1.64	2.03

Values are computed over the settled portion of each segment (second half of segment duration); multi-step segments 1–6 correspond to time windows (0–1, 1–15, 15–18, 18–27, 27–39, 39–48 h) while segment 1 (the initial ramp from ambient) is not reported.

**TABLE 2:** Environmental and CER metrics for the two biotic experiments, reported separately for the internal-growth (closed-mold) and external-growth (exposed) phases.

Variable	Multi-step run (multi-step)		Fixed-set-point (25 °C/45% RH)	
	Internal (0–2.8 days)	External (2.8–4.5 days)	Internal (0-2.8 days)	External (2.8–3.5 days)
Duration (d)	2.8	1.7	2.8	0.7
T2 mean ± SD (°C)	26.12 ± 1.02	25.75 ± 1.41	25.60 ± 0.06	25.71 ± 0.09
RH mean ± SD (%)	46.7 ± 8.1	51.2 ± 8.0	44.5 ± 3.5	45.0 ± 2.7
CO_2_ mean (ppm)	905	729	586	581
CO_2_ peak (ppm)	1049	849	706	627
CER mean (mmol kg^–1^ h^–1^)	2.58	1.68	0.95	0.92
CER peak (mmol kg^–1^ h^–1^)	3.32	2.29	1.57	1.16
CER peak time (d from experiment start)	0.73	2.84	2.47	2.88

CER, CO_2_ evolution rate (Equation 1). OUR and respiratory quotient not reported, see section “3.2 Biotic controller validation and online growth monitoring.” Multi-step run external phase duration reflects 4.5 days total run length; fixed-set-point external phase reflects the usable 3.5 days window preceding a 19 h logging outage.

**FIGURE 3:**
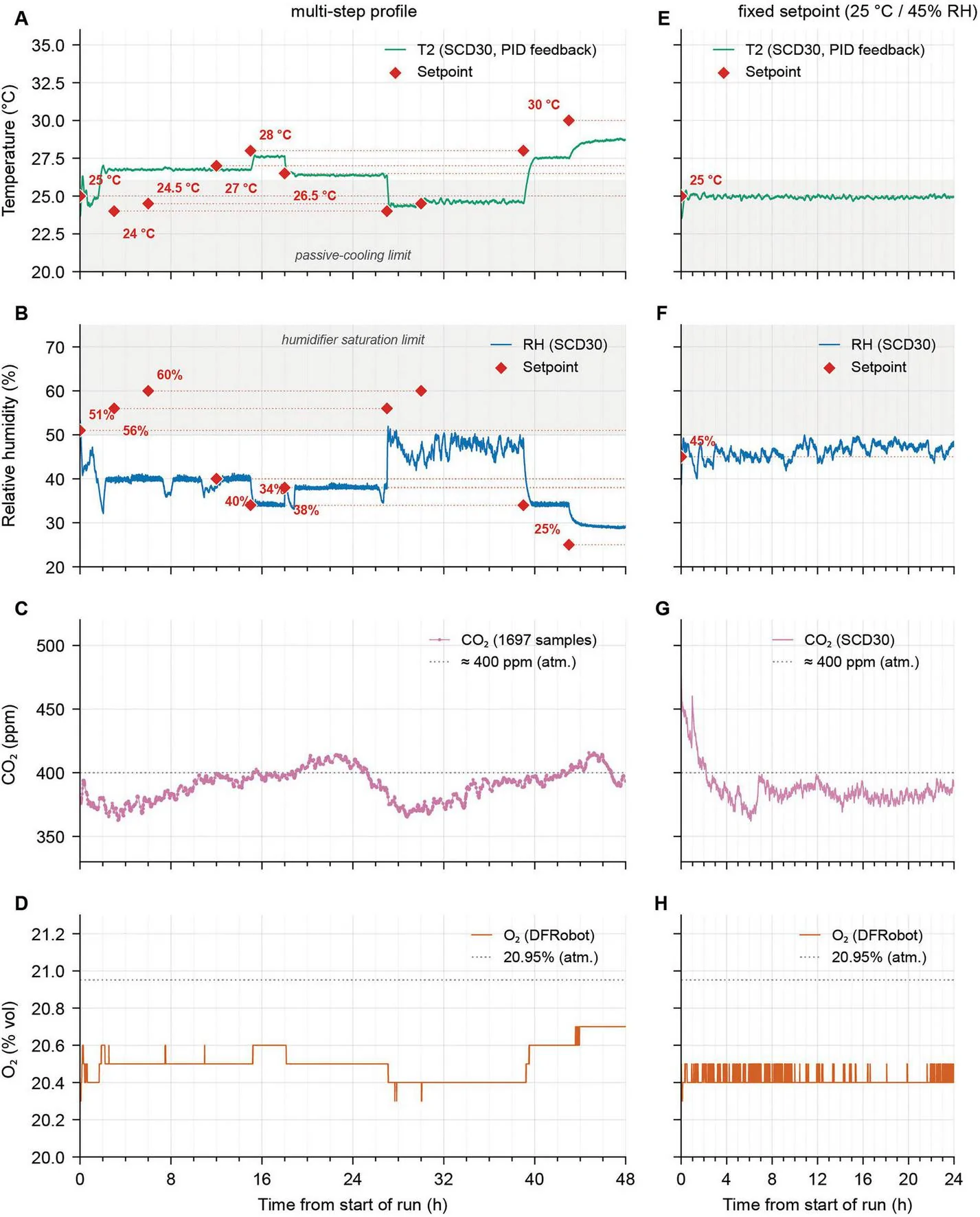
Abiotic controller validation of the tray bioreactor. **(A–D)** Multi-step abiotic run with programmed temperature and relative-humidity changes, including a terminal stress segment. **(A)** Temperature measured by the control sensor (T2), **(B)** relative humidity, **(C)** head-space CO_2_, and **(D)** head-space O_2_. Shaded regions indicate setpoints outside the practical operating envelope under the tested configuration. **(E–H)** Fixed-set-point abiotic run at 25 °C and 45% RH showing **(E)** temperature, **(F)** relative humidity, **(G)** head-space CO_2_, and **(H)** head-space O_2_. The results show stable closed-loop regulation within the controllable range and near-atmospheric gas composition throughout abiotic operation.

### 3.2 Biotic controller validation and online growth monitoring

The same two-experiment design was then applied under live fermentation conditions. This allowed us to assess controller performance in the presence of metabolic disturbances and to demonstrate online respirometric monitoring. The hemp-shive substrate described in section “2.5 Biological materials and fermentation protocol” was incubated in the bioreactor over a 4.5 days protocol consisting of a 2.8 days internal-growth phase (closed mold) followed by a 1.7 days external-growth phase after opening the mold (Figure 4). Two setpoint schedules were evaluated in separate runs: a multi-step circadian-plus-stress profile extended throughout 4.5 days (multi-step run) and a constant hold at 25 °C/45% RH (fixed-set-point). The CER was continuously computed from head-space CO_2_ according to Equation 1. OUR and respiratory quotient are not reported because the resolution of the electrochemical O_2_ sensor was insufficient to resolve the small O_2_ depletion signal under the present fermentation conditions; the raw O_2_ traces are provided in the Supplementary Figure 1. CER profiles are shown in Figures 4A, B. In the multi-step run, the CER increased from 1.9 mmol kg^–1^ h^–1^ in the first hour to a peak of 3.32 mmol kg^–^1 h^–1^ approximately 0.7 days, then gradually decreased during the internal phase to approximately 2.4 mmol kg^–1^ h^–1^ at the opening of the mold (*t* = 2.8 days). The transition between phases is visible in Figure 4 at *t* = 2.8 days: demoulding produced a transient drop in CER of approximately 0.5 mmol kg^–1^ h^–1^, accompanied by a concurrent step in head-space CO_2_ (Supplementary Figure 2), after which CER stabilized between 1.6 and 1.9 mmol kg^–1^ h^–1^ during the external phase. This response was recorded continuously because the part remained within the instrumented chamber after demoulding (section “2.5 Biological materials and fermentation protocol”).

**FIGURE 4:**
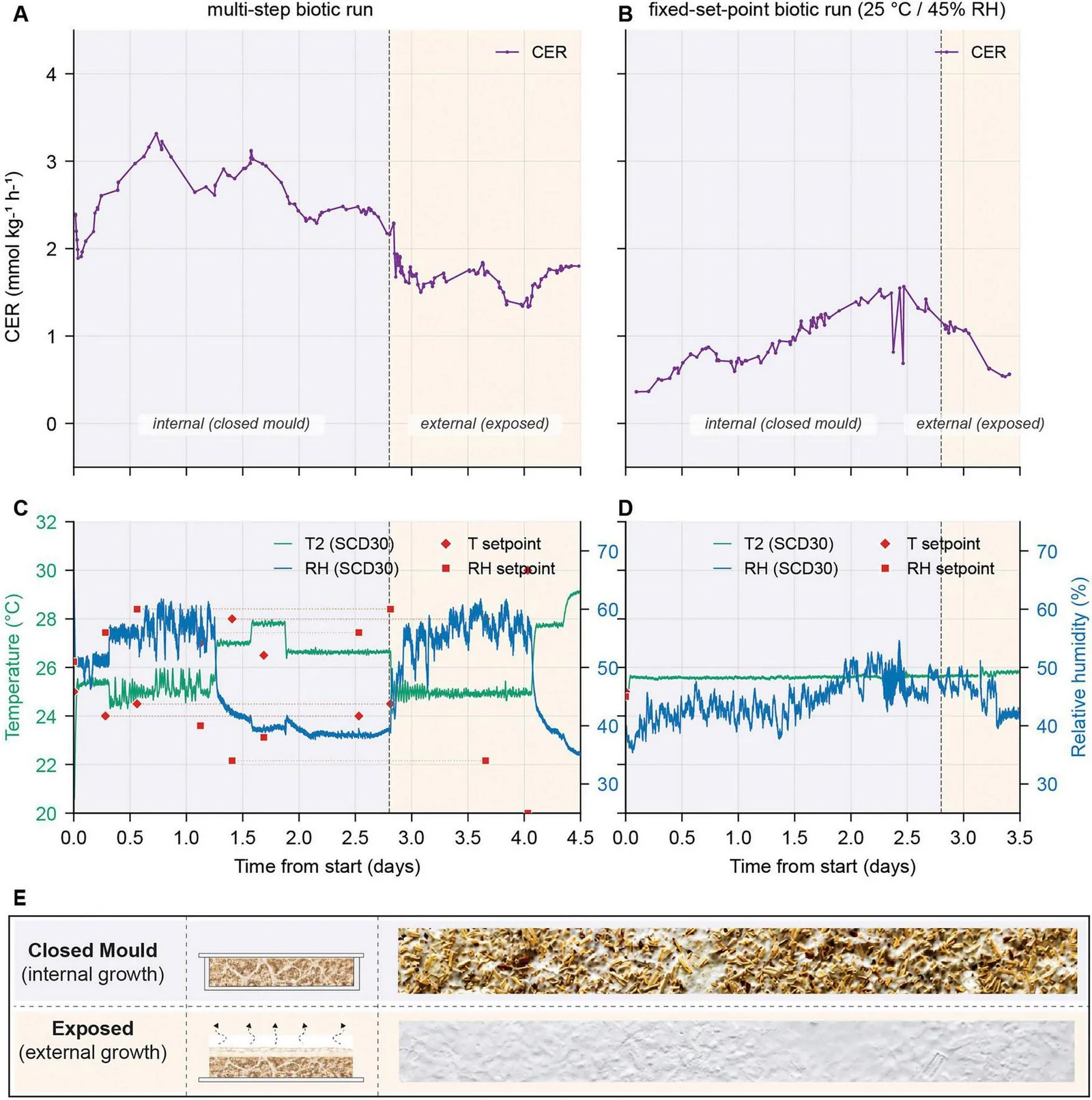
Biotic performance over a 4.5 days cultivation protocol comprising 2.8 days internal growth in a closed mold (lavender) followed by external growth after mold opening at *t* = 2.8 days (peach; dashed line). Left: multi-step run (extended circadian-plus-stress profile). Right: fixed-set-point run (25 °C/45% RH). **(A,B)** CER computed from head-space CO_2_ according to Equation 1. **(C,D)** Chamber temperature (T2, green, left axis) and relative humidity (blue, right axis) with temperature and RH setpoints indicated in red. **(E)** Schematic and representative surface textures for the two growth phases: closed mold/internal growth produced a substrate-exposed fibrous texture, whereas exposed/external growth after mold opening produced a denser, smoother mycelial surface skin. O_2_ traces are reported in Supplementary Figure 1 because sensor resolution was insufficient for quantitative OUR analysis. Phase-specific metrics are summarized in Table 2.

In the fixed-set-point run, the CER increased more gradually from 0.4 to a peak of 1.57 mmol kg^–1^ h^–1^ at approximately 2.5 days, then decreased throughout the internal phase and remained below 0.7 mmol kg^–^1 h^–1^ during the external phase. The phase-averaged CER was 2.58 mmol kg^–1^ h^–1^ during internal growth and 1.68 mmol kg^–1^ h^–1^ during external growth in the multi-step run, compared to 0.95 and 0.92 mmol kg^–1^ h^–1^, respectively, in the fixed-set-point run. The environmental control during fermentation remained comparable to that observed under abiotic conditions (Figures 4C, D). In the multi-step run, T2 followed the programmed temperature profile throughout the full 4.5 days period, with the same operating limits identified in section “3.1 Abiotic controller validation”: the set points below approximately 26 °C remained above the target, while the terminal high-temperature segment was below the upper thermal limit. In the fixed-set-point run, T2 remained stable at 25.6 °C ± 0.08 °C throughout the usable recording window. Relative humidity tracked the schedule closely at setpoints ≤ 40% in both runs, while the higher set-points in the multi-step profile again approached the upper RH ceiling identified in the abiotic experiments.

### 3.3 Multi-unit synchronization

To assess the transferability of the control architecture beyond a single chamber, the bioreactor platform was deployed as a four-unit array operated in a master–slave configuration. In this arrangement, one primary unit distributed setpoints, elapsed process time, and recipe-stage information to three connected secondary units over TCP/IP, while each reactor retained local sensing, actuation, and feedback control. The array combined two larger enclosures 1,000 × 1,000 × 500 mm (L × W × H) in the rear row with two smaller enclosures 1,000 × 500 × 500 mm (L × W × H) in the front row, demonstrating that the same control concept could be applied across chambers of different dimensions within one coordinated system (Figure 5). The resulting setup enabled simultaneous multi-unit operation under a shared supervisory schedule while preserving local environmental regulation at the individual reactor level. This array-level implementation establishes the feasibility of parallelized, synchronized cultivation for replicated experiments and future scale-out studies of mycelium-based composite fermentation.

**FIGURE 5:**
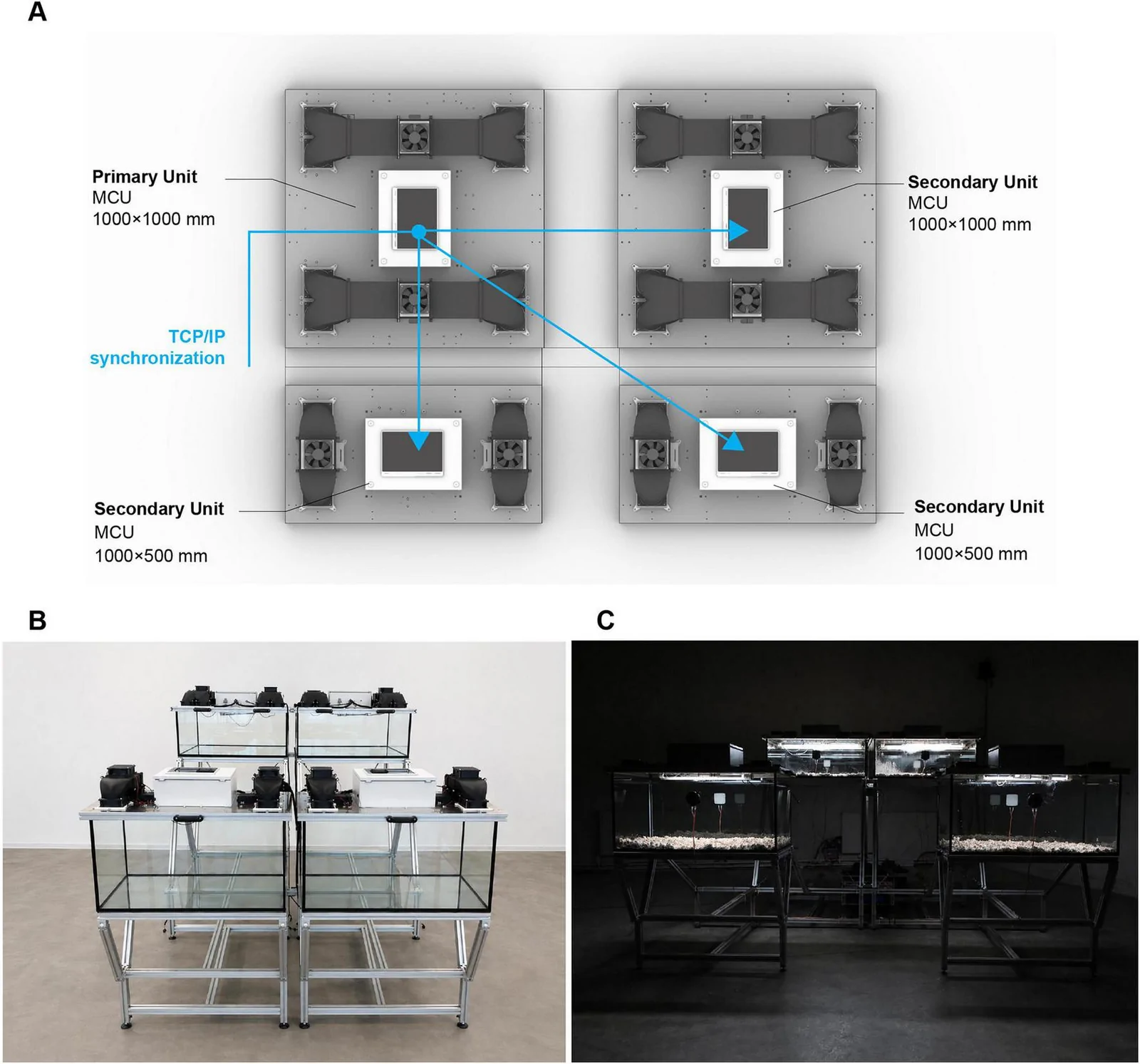
**(A)** Top-view schematic of the four-unit tray-bioreactor array operated in a master–slave configuration. The two rear units have enclosure dimensions of 1,000 × 1,000 × 500 mm (L × W × H), whereas the two frontal units have enclosure dimensions of 1,000 × 500 × 500 mm (L × W × H). One primary unit distributes setpoints, elapsed process time, and recipe-stage information to three secondary units over TCP/IP, while each reactor retains local sensing, actuation, and closed-loop environmental regulation. **(B)** Documentary view of the assembled four-unit array, showing the modular arrangement of chambers, lids, and control hardware. **(C)** Array in operation during synchronized incubation, illustrating simultaneous multi-unit deployment of the platform under active cultivation conditions. The array configuration extends the single-reactor control concept to coordinated multi-reactor experiments and replicated fermentation trials.

## 4 Discussion

### 4.1 Closed-loop environmental regulation during abiotic and biotic operation

The present study demonstrates that the tray bioreactor can maintain stable closed-loop regulation of temperature and relative humidity during both abiotic validation and live solid-state fermentation. Under abiotic conditions, the system regulated temperature reliably across the practically reachable range, with steady-state error below 0.5 °C, RMSE values between 0.13 °C and 0.46 °C, and re-establishment of new setpoints within approximately 30 min during the multi-step run. In the fixed-set-point experiment, the temperature stabilized at 24.92 °C ± 0.08 °C against a 25 °C target. Relative humidity showed similar good regulation at lower setpoints, with RMSE values of 0.24%–0.80% RH for segments at or below 40% RH, while higher humidity targets revealed an upper practical limit of approximately 47%–50% RH under the tested configuration. Together, these data define a practical operating envelope of roughly 26 °C-29°C and 25%–50% RH for the present hardware setup. A central outcome of the biotic experiments is that environmental regulation remained comparable in the presence of fungal metabolism. In the multi-step run, the chamber temperature followed the programmed profile throughout the 4.5 days cultivation period, while in the fixed-set-point run T2 remained stable at 25.6 °C ± 0.08 °C throughout the usable recording window. Relative humidity again tracked the schedule well at lower setpoints and approached the same upper ceiling observed during abiotic testing. This consistency between abiotic and biotic performance is important because it indicates that the control architecture remains functional under the thermal and moisture disturbances generated by live colonizing substrate. In the context of mycelium-based composites, where local environmental deviations can translate into heterogeneous growth and corresponding material defects, this level of regulation represents a significant advance over static or weakly controlled incubation approaches.

### 4.2 Online CO_2_ monitoring and CER as indicators of process state

A second major contribution of the platform is the integration of continuous gas-phase monitoring as an online readout of biological activity. The abiotic experiments established that, in the absence of metabolism, the head-space CO_2_ and O_2_ remained close to atmospheric values, with CO_2_ fluctuating between 365 and 415 ppm in the multi-step run and stabilizing at 383 ± 5 ppm in the fixed-set-point run after an initial transient. This baseline is critical because it shows that the gas-phase response measured during biotic runs cannot be attributed to chamber artifacts alone. Against this abiotic baseline, live fermentation produced clearly distinct CO_2_ and CER signatures. In the multi-step run, the CER increased from 1.9 mmol kg^–1^ h^–1^ within the first hour to a peak of 3.32 mmol kg^–1^ h^–1^ at approximately 0.7 days, then decreased to 2.4 mmol k^–1^ h^–1^ at the opening of the mold and stabilized between 1.6 and 1.9 mmol kg^–1^ h^–1^ during the external-growth phase. In the fixed-set-point run, CER rose more gradually from 0.4 to a peak of 1.57 mmol kg^–1^ h^–1^ at approximately 2.5 days and remained below 0.7 mmol kg^–1^ h^–1^ during the external phase. The Phase-averaged CER was correspondingly higher in the multi-step run than in the fixed-set-point run, both during internal growth and during external growth. The head-space CO_2_ also rose well above the abiotic baseline, reaching 1,049 ppm in the multi-step run and 706 ppm in the fixed-set-point run. In addition, mold opening produced an immediate gas-phase response in both experiments, visible as a clear drop in CO_2_ and a concurrent shift in CER behavior. These results show that the platform can detect biologically meaningful transitions online throughout the material-forming protocol rather than only through endpoint inspection of the final composite. During live fermentation with *G. sessile*, environmental conditions remained tightly regulated in both experiments, while accumulation of CO_2_ and CER clearly distinguished biological activity from the abiotic baseline. These signals also resolved the transition from confined growth within the closed mold to outward growth after mold opening. Although the monitored cultivation period was limited to five days, this window captured the most informative phase of metabolic activity, with the highest and most dynamic CER values occurring during early colonization before the system approached a less variable phase. Because the primary objective of this study was to validate the bioreactor platform, these results are important mainly to show that the system can maintain controlled conditions while detecting biologically significant changes in the state of the fungal process. The difference between the two biotic runs further suggests that dynamic environmental trajectories may influence metabolic activity during composite formation. The extended circadian-plus-stress schedule yielded substantially higher CER values than the constant 25 °C/45% RH condition, particularly during the internal-growth phase. This observation should be interpreted with caution, as the present work compares a limited number of fermentation runs and does not isolate the independent effects of the temperature history, the humidity history, or the biological variability between batches. Nevertheless, the contrast between the multi-step and the fixed-set-point run demonstrates the experimental value of a system capable of imposing reproducible time-varying conditions while simultaneously recording the metabolic response. In this sense, the platform provides a basis for future studies that aim to relate environmental trajectories to growth kinetics, colonization dynamics, and final material properties.

### 4.3 Technical limitations and interpretation boundaries

At the same time, the results define clear technical limits that should be explicitly stated. The first is that the commanded setpoint space was broader than the practically reachable operating range. In the multi-step abiotic experiment, temperatures below approximately 26 °C remained 1.4 °C-2.7 °C above the target, and during the terminal stress segment, the system approached but did not fully reach the 30 °C setpoint. Similarly, higher humidity setpoints at or above 51% RH remained 11%–16% below the target, indicating saturation of the humidification capacity. These findings suggest that the main current limitation lies in the hardware capacity, rather than in the instability of the control logic itself. The second limitation concerns the gas-phase interpretation. Although the platform continuously recorded CO_2_ and O_2_, the electrochemical sensor O_2_ did not provide sufficient resolution under current fermentation conditions for a robust quantitative analysis of the OUR or respiratory quotient. The 0.1% vol. quantization step of the electrochemical cell exceeds the metabolic O_2_ depletion observed under these operating conditions, as shown in Supplementary Figure 1. Additionally, the measured CER was not cross-validated against an independent reference gas analyzer; therefore, the CER values reflect the calibrated SCD30 CO_2_ signal and the open-loop airflow calibration rather than a verified absolute rate. Independent validation of CER against a reference instrument is identified as future work. Therefore, the manuscript appropriately restricts quantitative physiological interpretation to CER and treats O_2_ traces as supplementary. That distinction is important to keep the claims proportional to the evidence: the current system supports online CO_2_-based physiological monitoring, but not yet the full respirometric characterization of fungal metabolism. Likewise, airflow regulation was implemented in open loop from a pre-characterized fan-speed calibration, while fresh-air exchange remained operator-defined rather than actively closed loop. For that reason, the reactor is best described as a platform with closed-loop temperature and humidity regulation, open-loop airflow control, and continuous gas monitoring, rather than as a fully closed-loop gas-composition control system.

### 4.4 Relevance for replicated experimentation and future scale-up

Beyond the single-reactor experiments, the four-unit primary–secondary array shows that the control concept can be extended to coordinated multi-reactor operation. In this configuration, a primary unit distributed setpoints, the elapsed process time, and recipe-stage information to three secondary units over TCP/IP, while each chamber retained its own local sensing, actuation, and feedback control. The array also included chambers of two different dimensions, demonstrating that the same control architecture can be transferred across differently sized enclosures within one synchronized experimental platform. This aspect is particularly relevant for the future development of mycelium-based composite fermentation. Process optimization in SSF is unlikely to depend on a single fixed incubation condition; rather, it will require repeated comparative experiments across environmental schedules, chamber geometries, and substrate formulations. The demonstrated multi-unit architecture, therefore, adds practical value beyond the single-reactor validation because it provides a route toward replicated and synchronized studies with higher throughput and improved experimental comparability. The present platform also operated within clear constraints that define the boundaries of its current use. The reachable environmental envelope was limited by the cooling and humidification capacity of the hardware, airflow was regulated in open loop rather than under closed-loop control, and the electrochemical oxygen sensor lacked the resolution required for quantitative OUR and RQ analysis. Building on this platform, future work should address these constraints by extending the controllable temperature and humidity range, implementing closed-loop regulation of fresh-air exchange or CO_2_ concentration, upgrading O_2_ sensing for reliable OUR and RQ analysis, and correlating online process signals with endpoint measures such as colonization uniformity, density distribution, and mechanical performance of the resulting composites. In this way, the present system should be understood as a foundational experimental platform for moving mycelium-based composite production from empirically managed incubation to reproducible and scalable bio-process control.

## Data Availability

The raw data supporting the conclusions of this article will be made available by the authors, without undue reservation.
