# Supplementary material for: A closed-loop tray bioreactor for online monitoring of mycelium-based composites
Source: Front Microbiol. 2026 Sep 23;17:1877723. doi: 10.3389/fmicb.2026.1877723 (PMC13642839; doi:10.3389/fmicb.2026.1877723)
Supplement: Supplementary file 1 [file Data_Sheet_1.PDF]

Supplementary Material

1 SUPPLEMENTARY METHODS

1.1 PID controller tuning and parameters

Both feedback loops described in Section 2.3 of the main manuscript were implemented as discrete-time PID controllers running on the Compute Module 4 (CM4) at a 1 Hz sampling rate. Controller gains were tuned manually through step-response trials in the empty enclosure, starting from a Ziegler–Nichols proportional-only estimate and refined to minimise overshoot during set-point changes within the practical operating envelope identified in Section 3.1 (26–29 °C, 25–50 % RH). The temperature loop modulates the 100 W positive-temperature-coefficient (PTC) heater through pulse-width modulation (PWM) at 1 kHz, while the humidity loop opens and closes a solenoid valve in front of the ultrasonic atomising nozzle. Anti-windup was implemented through clamping of the integral term whenever the actuator output saturated. The tuned controller parameters used in all experiments reported in this study are listed in Supplementary Table S1.

1.2 Fan-speed to airflow calibration

Volumetric airflow through the recirculation circuit was characterised in a one-time, off-line procedure prior to the experimental campaign, as described in Section 2.2 of the main manuscript. Fan duty cycle was swept in 10 % increments from 30 % to 100 % PWM, and the air velocity at the exhaust of the recirculation HEPA filter was measured with a calibrated thermal anemometer (PCE-423N, PCE Instruments) at the centre of the duct cross-section. At each set point, the velocity was averaged over a 60 s window after a 30 s stabilisation interval, and converted to a volumetric flow rate using the measured cross-sectional area of the duct. The resulting fan-speed-to-flow relationship was approximately linear above 40 % PWM and was fitted with a third-order polynomial used as the open-loop look-up curve in subsequent runs. The anemometer was not used as an in-process sensor during fermentation, and recirculation does not contribute to net gas exchange, as discussed in Section 2.4.

1.3 Substrate preparation and pre-incubation

The substrate composition and inoculation protocol summarised in Section 2.5 of the main manuscript are reported here in extended form for reproducibility. Hemp shives (maximum particle length 4 mm) were rehydrated to a moisture content of 62% (w/w) and supplemented with wheat bran 5% (w/w) as an additional source of carbon and nitrogen and calcium sulphate dihydrate 1% (w/w) as a pH buffer and structural conditioner. The wetted substrate was packed into autoclavable polypropylene bags fitted with a 0.2 µm microporous filter patch and sterilised at 121 °C for 15 min at 103 kPa absolute pressure. After cooling to room temperature in a laminar-flow cabinet, millet-grain spawn of *Ganoderma sessile* was added aseptically at 16% (w/w) relative to the dry mass of the substrate and homogenised manually inside the bag. The inoculated substrate was incubated at approximately 26 °C and 60 % relative humidity for 5 days to allow initial colonisation before transfer to the bioreactor enclosure.

1.4 Multi-unit synchronisation protocol

The master–slave architecture introduced in Sections 2.3 and 3.3 of the main manuscript was implemented as a lightweight TCP/IP message protocol on the local network, with the master unit acting as a server on

a fixed port and each secondary unit subscribing as a client. The master broadcasts the active set-points (temperature, relative humidity, target fan duty cycle), the elapsed process time, and the current recipe-stage identifier at 1 Hz; secondary units acknowledge each broadcast and apply the received reference values to their own local PID loops. Each unit retains independent sensing, actuation, and feedback control, so loss of network connectivity reverts a secondary unit to the most recent valid set-point rather than interrupting environmental regulation. This design ensures that all reactors in the array follow the same supervisory schedule while local disturbances (e.g., mould opening, transient heater saturation) are rejected at the chamber level. The protocol was validated in the four-unit deployment described in Section 3.3.

1.5 Respirometric estimation of specific growth rate

The platform implements a stoichiometric framework for estimating the instantaneous specific growth rate $(\mu,\ \mathrm{h}^{-1})$ from the $\mathrm{O_2}$ uptake rate (OUR).The framework follows the relationship for aerobic fungal biomass synthesis, in which biomass formation is coupled to oxygen consumption through a biomass-on-oxygen yield coefficient (Torres-Mancera et al. (2018)):

$$\mu = \frac{\mathrm{OUR} \cdot Y_{\mathrm{O_2}}}{X} \tag{S1}$$

Two parameters in Eq. (S1) are not directly accessible in the present system. First, $Y_{\mathrm{O_2}}$ is strain- and substrate-specific, and no value has been reported for *Ganoderma sessile sp.* under solid-state fermentation. Reported coefficients for other filamentous fungi span a wide range — for example, approximately $0.78\ \mathrm{g\,biomass}\ \mathrm{g}^{-1}\ \mathrm{O_2}$ for *Rhizopus oligosporus* (Thanh and Nout (2002)) and approximately $2.84\ \mathrm{g\ biomass}\ \mathrm{g}^{-1}\ \mathrm{O_2}$ for *Aspergillus niger* (Saucedo-Castañeda et al. (1990), reported in energy-balance studies) — and in both cases the authors note that the coefficient is not constant over the course of a fermentation. Adopting a value from a different genus and substrate would therefore introduce an unquantified systematic error. Second, X cannot be measured directly in SSF because the mycelium and the lignocellulosic substrate are physically inseparable, and is conventionally inferred from the respirometric signal itself, creating a dependence on the same $\mathrm{O_2}$ measurement.

For these reasons, and because the $\mathrm{O_2}$ depletion under the present fermentation conditions fell below the 0.1% (v/v) quantization of the electrochemical sensor (Supplementary Fig. S1), Eq. (S1) was not evaluated in this study and is documented here as the basis for future growth-rate estimation once a strain-specific $Y_{\mathrm{O_2}}$ and a higher-resolution $\mathrm{O_2}$ sensor are available. Quantitative interpretation in the main text is restricted to CER (Eq. 1), which is derived from the $\mathrm{CO_2}$ signal and is independent of $Y_{\mathrm{O_2}}$ and X.

CER values were computed online from the raw SCD30 $\mathrm{CO_2}$ signal after applying a trailing moving-average filter with a 2-min window (N=60) samples at a 2-s sampling interval) to suppress sensor noise. The per-chamber head-space volumes V_e used in Eq. (1) were 0.2 m^3 for the smal enclosure and 0.4 m^3, for the large enclosure. These values were calculated from the internal enclosure dimensions after subtracting the displacement volumes of the lid and tray.

2 SUPPLEMENTARY TABLES AND FIGURES

REFERENCES

Saucedo-Castañeda, G., Gutiérrez-Rojas, M., Bacquet, G., Raimbault, M., and Viniegra-González, G. (1990). Heat transfer simulation in solid substrate fermentation. *Biotechnology and Bioengineering* 35, 802–808. doi:10.1002/bit.260350808

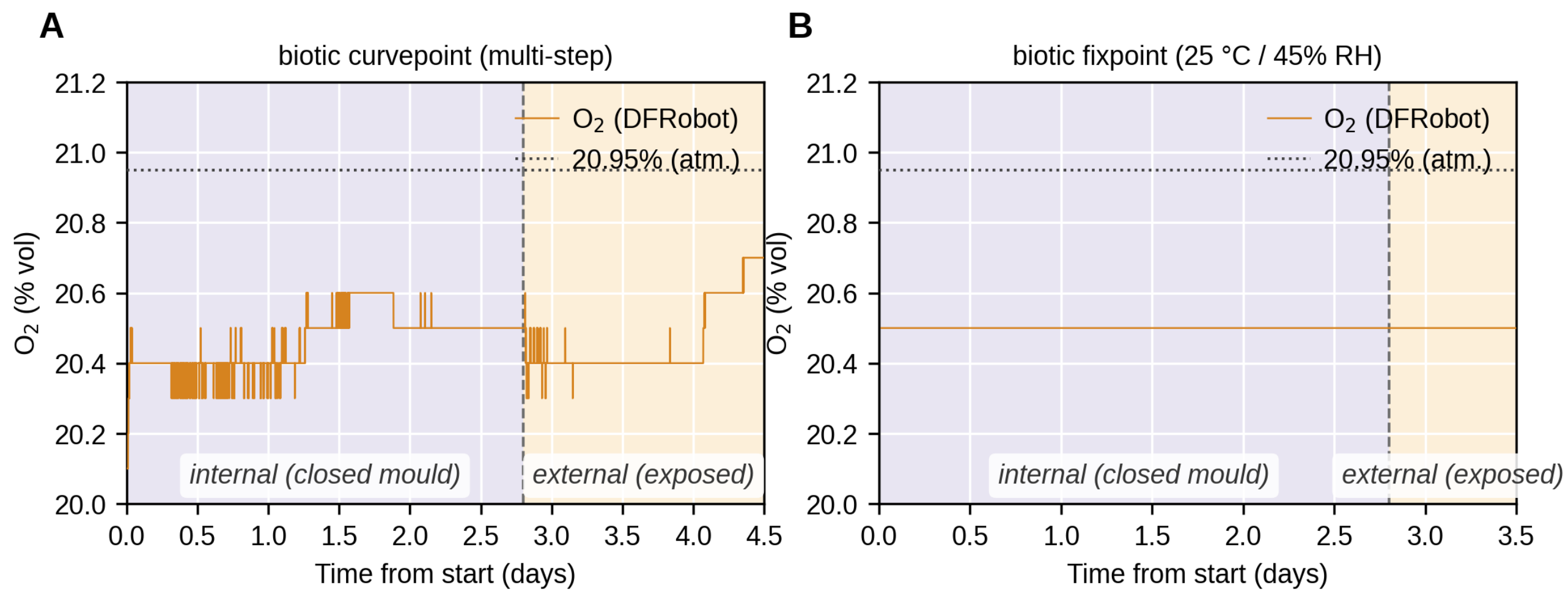

Figure S1. **H**ead-space O_2 concentration recorded by the DFRobot Gravity electrochemical sensor during the two biotic experiments. **(A)** multi-step run (extended circadian-plus-stress profile, 4.5 d). **(B)** fixed-set-point run (constant 25 °C and 45 % RH; 3.5 d usable window before a logging outage). Lavender shading indicates the internal-growth phase in the closed mould (0–2.8 d); peach shading indicates the external-growth phase after mould opening ($\geq$ 2.8 d, dashed line). The dotted reference at 20.95 % (v/v) marks the atmospheric value. Both traces show the systematic offset of approximately 0.4–0.5 % below atmospheric noted under abiotic conditions in Section 3.1, together with the discrete 0.1 % (v/v) quantisation imposed by the resolution of the electrochemical cell. In the multi-step run, a small depression in the first day coincides with the CER peak at 0.7 d, but the amplitude ($\leq$ 0.2 % (v/v)) is at the limit of sensor resolution. In the fixed-set-point run, the metabolic signal does not exceed the quantisation step at any point in the 3.5 d window. These traces therefore document the gas-phase O_2 measurement but cannot support quantitative OUR or respiratory-quotient analysis; physiological interpretation in the main manuscript is restricted to CER (Section 3.2 and Section 4.3).

Table S1. PID controller parameters used for closed-loop temperature and relative-humidity regulation. Gains apply to discrete-time PID controllers running at a sampling rate of 1 Hz on the reTerminal CM4. Feedback signals are described in Section 2.3 of the main manuscript.

**Loop**	**Feedback sensor**	K_p	K_i (s^{-1})	K_d (s)	**Output range**	**Anti-windup**
Temperature	Mean of T_1 and T_2	12.0	0.020	30	0–100 % PWM	Integral clamping
Relative humidity	SCD30 RH	4.5	0.010	0	Valve ON/OFF	Integral clamping

Thanh, N. and Nout, M. (2002). Rhizopus oligosporus biomass, sporangiospore yield and viability as influenced by harvesting age and processing conditions. *Food Microbiology* 19, 91–96. doi:10.1006/fmic.2001.0450

Torres-Mancera, M. T., Figueroa-Montero, A., Favela-Torres, E., Rosales-Zamora, G., Nampoothiri, K. M., and Saucedo-Castañeda, G. (2018). Online Monitoring of Solid-State Fermentation Using Respirometry. In *Current Developments in Biotechnology and Bioengineering* (Elsevier). 97–108. doi:10.1016/B978-0-444-63990-5.00006-2

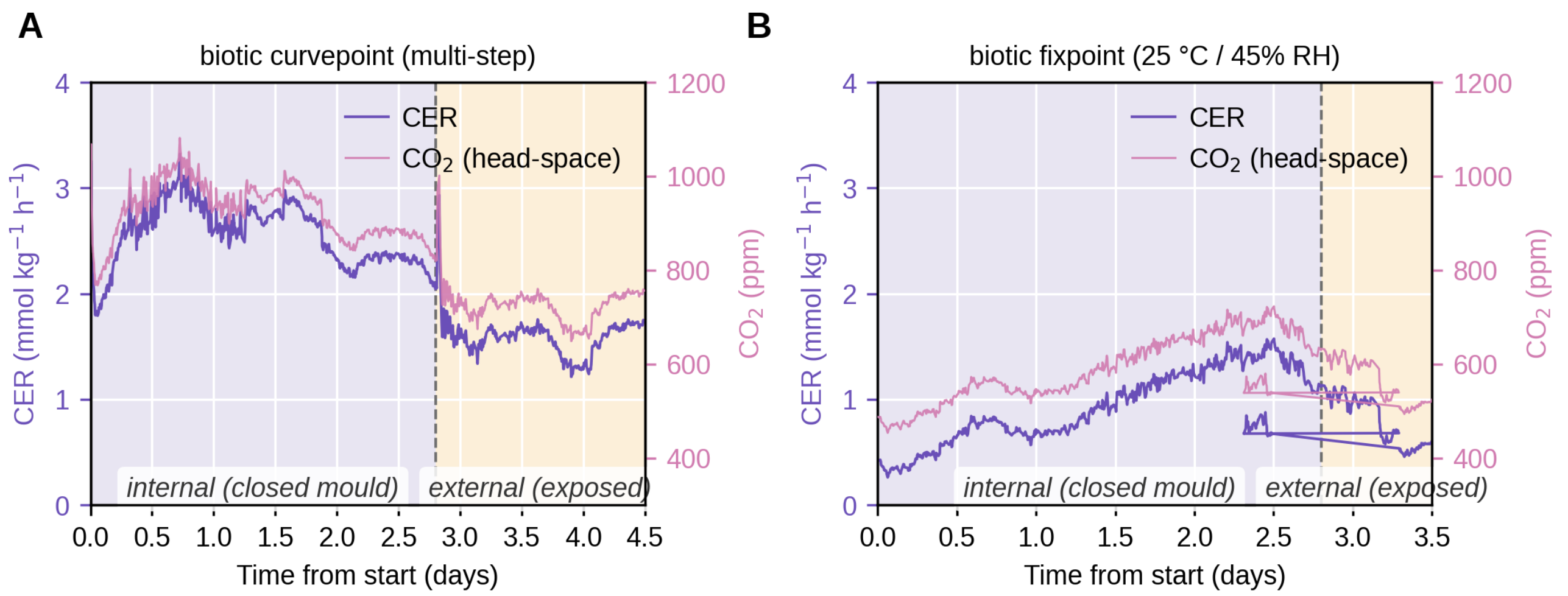

Figure S2. **C**ontinuous CER and head-space CO_2 during the two biotic experiments, computed from the raw SCD30 measurements per Equation 1 of the main manuscript. **(A)** multi-step run (extended circadian-plus-stress profile, 4.5 d). **(B)** fixed-set-point run (constant 25 °C / 45 % RH; 3.5 d usable window before logging outage). The CER signal (left axis, purple) is derived directly from the smoothed CO_2 trace (right axis, pink) using the procedure described in Supplementary Methods Section 1.5; isolated single-sample artefacts at the start of the logging outage in panel B were excluded. The visible coupling between the two signals confirms that the CER curves shown are quantitatively traceable to the raw CO_2 measurements without additional processing. The mould-opening transition at t = 2.8 d in panel A produces the transient drop of ≈ 0.5 mmol kg^{-1} h^{-1} described in Section 3.2.

Table S2. Extended per-segment controller-tracking metrics for the multi-step abiotic run. Values are reported for both the settled portion of each segment and the full segment, including the transient.

**Segment**	t **(h)**	T_{set} **(°C)**	RH_{set} **(%)**	**Settled** T **mean ± SD (°C)**	**Full-segment** T **RMSE (°C)**	**Settled RH mean ± SD (%)**	**Full-segment RH RMSE (%)**	**Settling time (min)**
1	1–15	27.0	40.0	26.67 ± 0.37	0.61	39.37 ± 1.46	1.92	≈ 30
2	15–18	28.0	34.0	27.60 ± 0.07	0.18	34.20 ± 0.28	0.45	≈ 25
3	18–27	26.5	38.0	26.38 ± 0.05	0.16	37.65 ± 1.11	2.71	≈ 30
4	27–39	24.5	48.0	24.55 ± 0.14	0.20	47.12 ± 1.84	2.14	≈ 35
5	39–48	28.0	30.0	28.02 ± 0.70	0.84	31.69 ± 2.71	3.41	≈ 40

Notes. Settled values were computed over the second half of each segment, consistent with Table 1. Full-segment RMSE includes the transient following each set-point change. Settling time is defined as the time required for T_2 to enter and remain within ±0.3 °C of the new set point.

Table S3. Mean headspace CO_2 and O_2 concentrations during the two abiotic experiments, computed over the full duration of each run after excluding the initial 2 h chamber-equilibration period. The values establish the abiotic baseline against which the biotic CER signal in Section 3.2 is interpreted.

Run	Duration (h)	CO_2 mean ± SD (ppm)	CO_2 range (ppm)	O_2 mean ± SD (vol.%)	O_2 range (vol.%)
Multi-step abiotic	48	391 ± 13	363–416	20.51 ± 0.09	20.3–20.7
Fixed-set-point abiotic	24	384 ± 7	362–408	20.45 ± 0.03	20.4–20.5

Notes. The systematic offset of approximately 0.4–0.5 vol.% below the theoretical atmospheric O_2 concentration of 20.95 vol.% is consistent with the calibration of the DFRobot Gravity electrochemical sensor and is discussed in Section 4.3 of the main manuscript. Spurious zero readings recorded during occasional sensor dropouts, accounting for less than 0.1% of all measurements, were excluded from the O_2 statistics.
